# Effects of plyometric training performed on different surfaces and with different types of footwear on the neuromuscular performance of team-sport athletes: A systematic review

**DOI:** 10.5114/biolsport.2025.150037

**Published:** 2025-04-28

**Authors:** Gastón R. Sanchez-Ottado, Konstantinos Spyrou, Lucas A. Pereira, Pedro E. Alcaraz, Santiago Zabaloy, Irineu Loturco, Tomás T. Freitas

**Affiliations:** 1 UCAM Research Center for High Performance Sport, Catholic University of Murcia, Murcia, Spain; 2 Facultad de Deporte, UCAM Universidad Católica de Murcia, Murcia, Spain; 3 NAR—Nucleus of High Performance in Sport, Saõ Paulo, Brazil; 4 Faculty of Physical Activity and Sports, University of Flores, Buenos Aires, Argentina; 5 Department of Human Movement Sciences, Federal University of São Paulo, São Paulo, Brazil; 6 Department of Sport and Exercise, University of South Wales, Pontypridd, Wales

**Keywords:** Jump training, Athletic performance, Plyometrics, Power, Speed, Strength

## Abstract

This systematic review aimed to analyze the effects of plyometric training (PT) performed on different surfaces and with different types of footwear on the neuromuscular performance of team-sport athletes, and to properly delineate the role of these specific factors (i.e., surface type and footwear) on PT outcomes. A systematic search was conducted according to the preferred reporting items for systematic reviews and metaanalyses guidelines using PubMed, Scopus, and Web of Science for articles published before May 2024. From the total of 2832 articles, 35 met the inclusion criteria for the systematic review. The results indicate that sand surfaces seem to be more effective than other surfaces in increasing neuromuscular performance. Specifically, studies that investigated the intervention process found significant improvements in performance metrics after plyometric sand training. In terms of acute effects, the results were diverse and inconclusive, with no clear pattern of evidence. Despite presenting lower improvements overall, rigid surfaces required a lower number of contacts compared to other types of surfaces to achieve similar performance gains. PT in water is also recommended to promote neuromuscular adaptations. Regarding footwear, minimalist and rigid options were found to lead to higher improvements in various neuromuscular performance variables, likely due to enhanced energy efficiency and stability during PT sessions. The included studies indicated that PT on sand is highly effective for improving neuromuscular adaptations. However, training on rigid surfaces is more time-efficient, while aquatic surfaces are also recommended. Additionally, minimalist or rigid footwear acutely improves various athletic performance variables. Overall, when designing a PT program, it is crucial to consider both the surface and footwear to maximize neuromuscular adaptations.

## INTRODUCTION

Resistance training is of considerable importance in the preparation of high-level athletes due to its effectiveness in enhancing physical performance and potentially reducing the risk of injury [1]. Engaging in strength training programs that combine high-load, low-velocity exercises (e.g., heavy squats) with low-load, high-velocity exercises (e.g., loaded and unloaded jumps) leads to improvements in both maximum strength and power [2]. Furthermore, strength is associated with other important physical capabilities, such as jumping, sprinting, and changing direction [1], which may transfer to fundamental team-sport skills such as turns, linear and multidirectional sprints [1]. To some extent, this may be explained by changes at both the neuronal level (i.e., activation, recruitment, and synchronization of motor units [2]) and at the morphological level (i.e., increased cross-sectional area and muscle pennation angles [2]) that occur following resistance training. Such adaptations clearly contribute to enhancing performance, potentially reducing injury rates [3], and expediting post-competition recovery [4, 5], thereby promoting athletes’ availability to train and compete [2].

Among various strength-power training methods, one commonly used approach by coaches in both team- and individual sports [6, 7] to increase fast force production is plyometric training (PT). PT primarily involves numerous forms of jumping [8] and consists of muscle stretching (i.e., eccentric action) followed by an explosive push-off (i.e., concentric action) [9], a process known as the “stretch-shortening cycle” (SSC) [10]. This method mimics the specific high-intensity, short-duration actions, typically encountered in competitive settings, potentially increasing the transfer effect between jump tasks and sport-specific performance [6, 11, 12]. PT has proven effective in improving several physical qualities, including maximum strength, jumping ability, agility, sprinting, running economy, and consequently, endurance [6]. Moreover, PT requires little equipment and limited space, making it a cost-effective solution for improving the physical performance of team-sport athletes [13]. In addition, various forms of PT can be performed both on the field and in the weight room. However, the effects of PT executed on different surfaces remain relatively unclear in this population. This is partly due to inadequate reporting in the literature, as noted in a recent systematic review [14] on PT for soccer players, in which 64% of the included studies did not specify the type of surface used.

Of note, in the review by Ramírez-Campillo et al. [14], among the studies that specified the training surface, grass was the most commonly used (reported in 22% of the included studies), possibly due to its association with the surface on which soccer players usually train [14]. Indeed, grass appears to be one of the most effective surfaces for developing power, sprinting, agility, and strength in soccer players [15]. However, PT on softer surfaces, such as sand, may offer similar improvements in athletic performance (e.g., sprinting or squat jump [SJ] abilities) as those achieved on grass, while causing less muscle soreness [16]. These adaptations may be attributed to the fact that PT on sand requires a longer duration concentric pushing phase, which is caused by the surface’s absorption of elastic energy [16, 17]. Conversely, on harder surfaces like grass or parquet, the most noticeable improvements are suggested to occur in the countermovement jump (CMJ) [16, 17], possibly due to the stiffness of the surface, which allows for greater engagement of the SSC by preventing the loss of elastic energy that typically occurs on sand. These results seem to indicate that soft surfaces (e.g., sand) may be more favorable for improving muscle contractile properties, while hard surfaces (e.g., grass or parquet) may be more conducive to enhancing SSC function [16, 17]. Supporting this notion, a 7-week PT protocol conducted on a wooden floor resulted in greater improvements in the reactive strength index (+29.7%; measured from a 20-cm drop jump [DJ]) compared to when performed on a softer mat of 3-cm thickness [18]. It is also worth noting that the softer surface negatively impacted change of direction (COD) ability, showing an increase of 2.4% in completion time [18]. Therefore, it remains unclear to what extent PT performed on a gym floor with a soft mat or directly on a soccer field can lead to similar adaptations.

Another factor that could influence neuromuscular adaptations following PT is the type of footwear used during training. Despite this, only a few studies [19–23] have specifically addressed this aspect. For example, LaPorta et al. [19] investigated different physical performance variables, comparing three types of footwear: athletic shoes, minimalist shoes, and barefoot (i.e., no footwear). The authors found that peak power during tests like the CMJ test and Bosco test was higher in the latter condition, and jump height was also higher when subjects jumped barefoot or with minimalist shoes compared to when using athletic shoes. One possible explanation provided by the authors was that the padding in athletic shoes may dissipate force, preventing it from being efficiently transferred to the ground [19]. Nevertheless, despite anecdotal evidence indicating a growing trend of barefoot PT among athletes, little is known about the influence of different types of footwear on the acute and longterm responses to this type of training.

Given the above and considering the current gap identified in the literature, this systematic review aimed to analyze the effects of PT performed on different surfaces (e.g., grass, concrete, wooden floor/parquet, sand, and water) and using different types of footwear (i.e., athletic shoes, minimalist shoes) or barefoot on neuromuscular performance in team-sport athletes. Additionally, it sought to properly delineate the role of specific factors (i.e., surface type and footwear) and their differential effects on PT outcomes. This systematic analysis of the current literature may be valuable for coaches and practitioners, providing crucial insights that allow the prescription of more effective PT programs in team-sport contexts, considering the nature of the surface and the type of footwear.

## MATERIALS AND METHODS

### Protocol and Registration

This review was conducted in accordance with the Preferred Reporting Items for Systematic Reviews and Meta-Analyses (PRISMA) guidelines [24]. The protocol for this systematic review was preliminarily submitted and published on the Open Science Framework, with the registration number 10.17605/OSF.IO/JGBVZ on 25^th^ April 2024.

### Data sources and searches

The search for the present systematic review was conducted across three different online databases, PubMed, Scopus, and Web of Science. The search was restricted to original articles written in English and published online, with a cutoff date of May 23, 2024. The following keywords were used in the search, combined with different Boolean operators, such as “AND” or “OR”: “athletes”, “football”, “soccer”, “team sports”, “player”, “plyometric”, “jump”, “surface”, “sand”, “hard”, “floor”, “grass”, “concrete”, “unstable surface”, “gym mat”, “water”, “shoes”, “barefoot”, “footwear”, “sprint”, “strength”, “repeated sprint ability”, “delayed onset muscle soreness”, “change of direction”, “agility”, “performance”. An example search strategy used in PubMed was: (Athletes OR football OR soccer OR “team sports” OR “team-sports” OR player$) AND (plyometric OR jump*) AND (surface OR sand OR hard OR floor OR grass OR concrete OR “unstable surface” OR “gym mat$” OR water) AND (jump OR sprint OR strength OR “repeated sprint ability” OR “delayed onset muscle soreness” OR “change of direction” OR agility OR performance). In addition, the references of relevant articles were reviewed to identify any additional studies that could be included in the review.

### Inclusion criteria

Studies were eligible for inclusion if they met the following criteria: 1) the sample consisted of male team-sport players; 2) a cross-sectional (i.e., assessing acute responses to training) and/or longitudinal study was designed; 3) players were aged ≥ 14 years, to avoid the potential influence of the maturational status of the athletes, which has been shown to affect the neuromuscular adaptations to PT [25–27]; 4) the study outcomes comprised one of the following physical performance variables: linear and curvilinear sprints, COD, vertical and horizontal jumps, muscular strength, or muscle damage and delayed onset muscle soreness (i.e., DOMS); 5) participants completed a PT program on different surfaces (e.g., grass, concrete, wooden floor/parquet, sand, and water) and/or using different types of footwear (e.g., standard, minimalist) or barefoot; and 6) the study was published in English.

### Exclusion criteria

Studies were excluded if: 1) the interventions were performed on surfaces not suitable for sports practice (i.e., unstable surfaces); or 2) footwear comparisons did not involve differences in rigidity (e.g., collar type).

### Study Selection

The search strategy and study selection were performed by two authors (GSO and KS). Results were uploaded to a reference management software (Zotero, Virginia, USA), where duplicates were automatically removed. After duplicates were eliminated, an extensive review of all titles and abstracts was conducted by the same two authors (GSO and KS). The eligibility criteria mentioned above were strictly followed, and entries not related to the review’s topic were discarded. Any disagreements regarding study inclusion and exclusion that could not be resolved between the two authors were settled by a third party (TTF). The full version of the remaining articles was then reviewed, and all studies not meeting the inclusion criteria were excluded.

### Data extraction

One investigator (GSO) processed all data collected. Only data corresponding to the variables of interest (i.e., linear sprints of various distances [5–30 m], sprints incorporating a COD, vertical and horizontal jumps, muscular strength, muscle damage and DOMS) were extracted from each eligible study. Data were entered into a custommade Microsoft Excel sheet by one author (GSO), with two other authors (KS and TTF) verifying accuracy. The following data were retrieved: study information (i.e., author and year), participant details (i.e., sample size, age, height, weight), intervention characteristics (i.e., weeks, frequency, total of sessions, duration of sessions, exercises), variables of interest (i.e., linear and curvilinear sprints, COD, vertical and horizontal jumps, muscular strength, muscle damage and DOMS), tests or assessments performed, and acute responses and short- to long-term adaptations. A meta-analysis was not conducted due to the heterogeneous nature of the study designs and the inability to pool the data.

### Risk of bias

The assessment of bias risk was conducted independently by two authors (GSO and KS), with any discrepancies resolved through reanalysis. In cases where consensus could not be reached, a final decision was made by the senior author (TTF). The risk-of-bias tool for randomized trials (RoB 2) was used to evaluate risk of bias in the included studies. Moderate reliability, along with strong feasibility and validity of this instrument, has been previously reported [28]. The tool comprises five domains: 1) the randomization process; 2) deviations from intended interventions; 3) missing outcome data; 4) measurement of the outcome; and 5) selection of the reported result. Each domain was classified as having ‘low’, ‘high’, or ‘unclear’ risk of bias [28].

### Methodological quality assessment

The selected studies with intervention protocols were submitted to the PEDro [29] methodological quality scale. The PEDro checklist includes 11 items, but the first item is not rated, resulting in a maximum score of 10 and a minimum of 0. As in previous PT metaanalyses [30], studies scoring ≤ 3 points were considered as “poor quality”, 4–5 points as “moderate quality”, and 6–10 points as “high quality”. Two reviewers (GSO and KS) independently evaluated the methodological quality, resolving any disagreements through discussion with a third author (TTF).

For studies investigating acute effects, the “Quality Assessment Tool for Observational Cohort and Cross-Sectional Studies” (QATOCCS) [31] was used to evaluate the risk of bias based on 14 key criteria. Demerit points were assigned for every ‘No’, ‘Cannot Determine’ (CD), or ‘Not Reported’ (NR) response. Studies received an overall grade of ‘Good’ with two or fewer demerits, ‘Fair’ with three to six demerits, and ‘Poor’ with more than six demerits. Criteria marked as ‘Not Applicable’ (NA) did not incur a demerit. This grading method was established by consensus of the research team.

## RESULTS

### Search Results

**FIG. 1:**
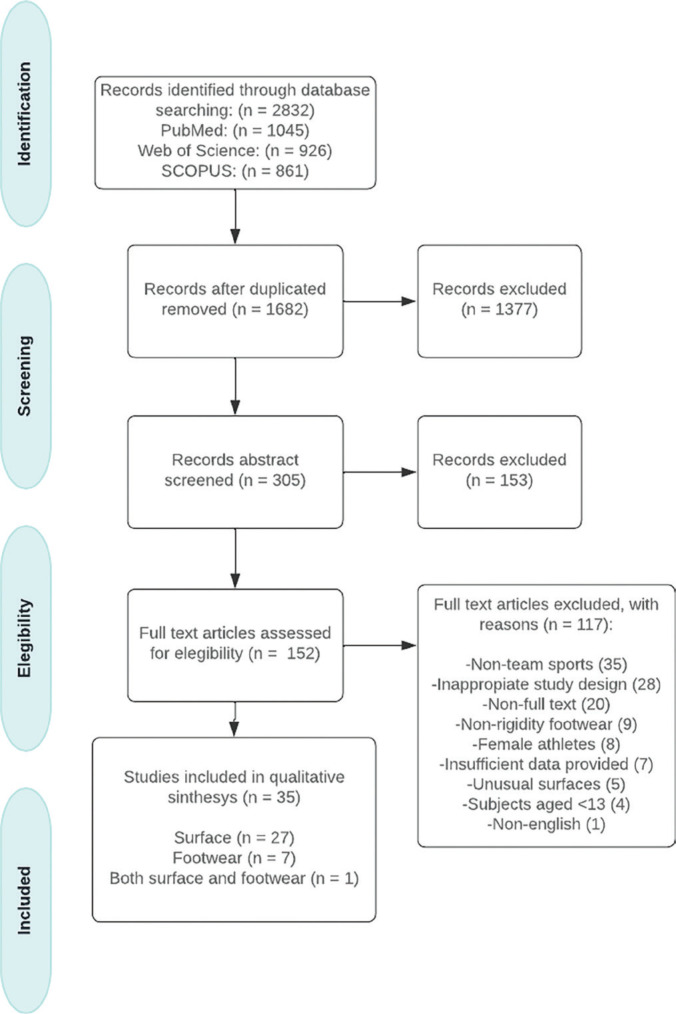
Flow diagram of the study identification and selection process.

Figure 1 shows the PRISMA flow chart of the search and selection process. A total of 2,832 studies were identified during the search phase. After removing duplicates, 1,682 publications were retained for the article selection process. During the title and abstract screening, 1,530 articles were excluded. The remaining 152 records were further examined based on the specified inclusion and exclusion criteria, and 117 records were subsequently rejected for the following reasons: 1) non-team-sports (n = 35), 2) inappropriate study design (n = 28), 3) lack of full text (n = 20), 4) lack of specification regarding footwear rigidity (n = 9), 5) female athletes (n = 8), 6) insufficient data (n = 7), 7) unusual surfaces (e.g., unstable surface) (n = 5), 8) subjects younger than 13 years old (n = 4), and 9) non-English language (n = 1). Finally, 35 studies were included in the systematic review: 28 focused on comparing different surfaces (17 intervention studies and 11 on acute effects), 8 analyzed different footwear, and 1 study addressed both categories.

### Methodological Quality and Risk of Bias

After applying the PEDro checklist to the studies with intervention protocols [16, 17, 32–46], eight studies scored > 6 points, indicating a high level of methodological quality. Additionally, nine studies attained a score of 4–5 points, indicating a moderate level of methodological quality (see Supplementary Material).

For the studies evaluating acute effects [21–23, 47–61], the QATOCCS was used to categorize studies as “good (0–2 demerits), fair (3–5 demerits), or poor (≥ 6 demerits)”. Nine studies [21–23, 49, 52–54, 58, 60] were rated as “fair”. The remaining nine studies [47, 48, 50, 51, 55–57, 59, 61] received 6 demerits and were therefore rated as “poor” (see Supplementary Material).

**FIG. 2:**
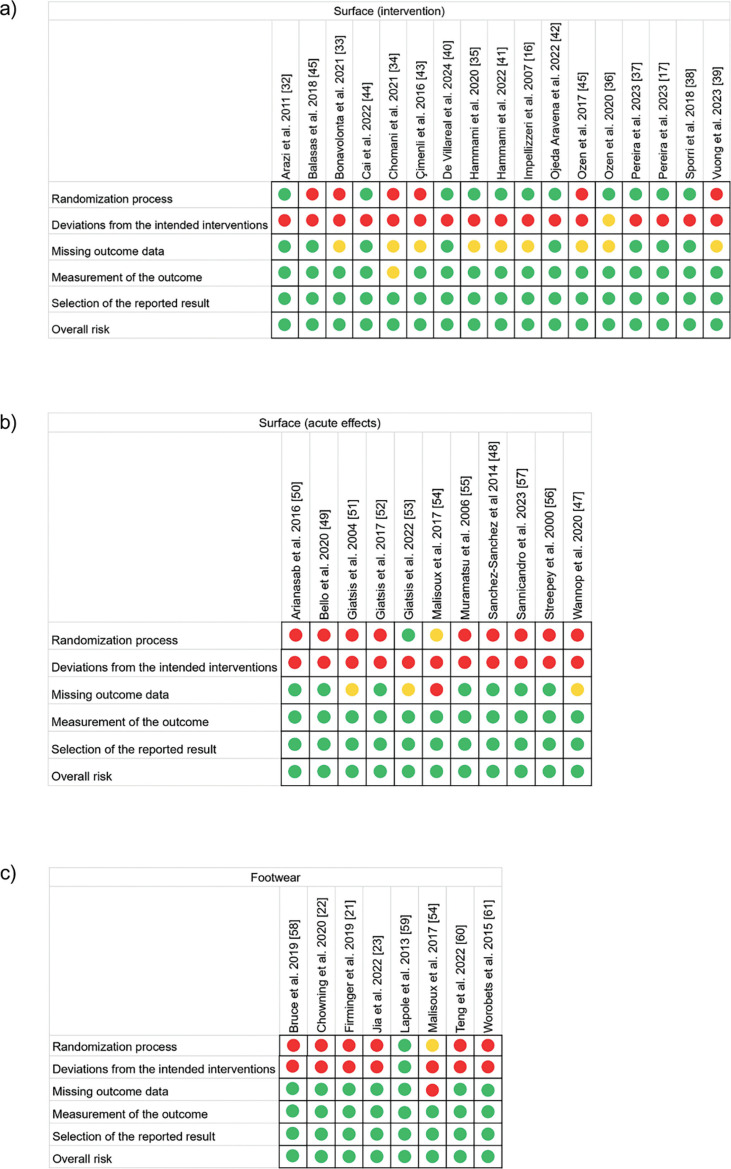
Risk of bias assessment for the studies investigating: (A) the short- to long-term effects of plyometric training programs performed on different surfaces; (B) the acute responses to plyometric training programs performed on different surfaces; (C) the effects of plyometric training programs performed with different footwear.

The results of the risk of bias assessment are shown in Figures 2. Overall, the randomization process was clearly described in 37% of the articles (intervention process surface [11/17], acute effects surface [1/11], and footwear [1/8]). This is primarily because several of the acute effects and footwear studies did not randomize their samples. The risk of bias due to deviations from the intended interventions was high in 94% of the articles, as participants were aware of their group, which is common in sport science research. There was a low risk of bias for missing outcome data in 63% of the studies, while the remaining 31% presented an unclear risk of bias. The risk of bias in outcome measurement was very low (97%) due to the validity of the evaluation instruments. Finally, all included studies presented a low risk of bias in the selection of the reported results.

### Surfaces

#### Short-term adaptations following plyometric training performed on different surfaces

Table 1 presents the results of the studies analyzing the effects of PT interventions performed on different surfaces on neuromuscular performance. Of the 35 studies included in the systematic review, 27 involved intervention protocols to assess the effects of different surfaces on performance outcomes. Among these, 12 studies measured sprint performance, 9 assessed COD speed, 16 evaluated vertical and horizontal jumps, 3 examined changes in muscular strength, and 2 studies investigated DOMS. These studies provide a comprehensive analysis of the impact of various surfaces on athletic performance following the PT interventions.

**TABLE 1:** Studies investigating the influence of training surface on short- to long-term adaptations induced by plyometric training.

Study	Study group	N	Age (years)	BM (kg)	Height (cm)	Wks	F	T	D (m)	Excercise type	Test	Response
Arazi et al. 2011 [32]	WATER	6	18 ± 0.60	75.6 ± 3.9	180.2 ± 4.5	8	3	24	40	Ankle jump	Muscular strength	Muscular strength: WATER ↑
	DRY-LAND	6	18.03 ± 1.38	67.5 ± 1	182.4 ± 7.2					Speed marching	36.5-m sprint	36.5-m sprint: WATER ↑, LAND ↑
	CONTROL	6	20.4 ± 0.64	60.2 ± 7.0	175.3 ± 4.6					SJ Skipping	60-m sprint	60-m sprint: WATER ↑, LAND ↑

Balasas et al. 2018 [45]	SAND RIGID	11	26.5 ± 3.3	84.6 ± 6.2	187 ± 5.0	12	4–6	48–72	90–120	NR	VJ	VJ: SAND ↑, RIGID ↑

Bonavolonta et al. 2021 [33]	GRASS	8	24.4 ± 5	NR	NR	7	2	14	25	Lateral 0.3 m hurdle jumps (3 to left and 3 to right) Sprinting 10 m. Horizontal jumps (3 to left and 3 to right) Hurdle jumps	5-m sprint 10-m sprint 20-m sprint VJ HJ Agility	5-m sprint: SAND ↑ 10-m sprint: SAND ↑ 20-m sprint: SAND ↑ VJ: SAND ↑ HJ: SAND ↑ Agility: ↔
	SAND	8										

Cai et al. 2022 [44]	WATER	10	20.4 ± 1.8	81.4 ± 8.9	185.2 ± 9.5	10	2	20	60	SJ VJ Jump and touch high Double-pedal jump Bend knees Jump with feet sideways Vertical on one foot Z-Jump Hop	Muscle strength VJ Muscle damage (serum CK) DOMS	Muscle strength: WATER ↑ VJ: WATER ↑ Muscle damage (serum CK) ↔ DOMS: ↔
	CONTROL	10										

Chomani et al. 2021 [34]	WATER	20	16.25 ± 1.0	61.03 ± 4.0	168 ± 3.0	8	2	16	NR	Jumps-in-Place Multiple hops and jumps Standing Jumps Box Drills Bounding Deep Jumps	NR	VJ: WATER ↑ HJ: WATER ↑ Agility: WATER ↑ Sprint: WATER ↑
	DRY-LAND	20										
	CONTROL	20										

Çimenli et al. 2016 [43]	WOOD	12	18–-24	73.7 ± 6.7	183.5 ± 7.3	8	3	24	50–60	NR	VJ	VJ: WOOD ↑, SYNTHETIC ↑
	SYNTHETIC	12		83.1 ± 14.8	185.4 ± 3.7						HJ	HJ: WOOD ↑, SYNTHETIC ↑
	CONTROL	12		79.7 ± 3.1	185.3 ± 3.8							

De Villareal et al. 2024 [40]	SAND	12	23.0 ± 4.9	79.1 ± 8.3	180.9 ± 6.0	6	3	18	40	SJ Skipping Double-leg hops Double-leg speed hops Side hops Alternative leg bounds 15- m sprint	15-m sprint VJ	15-m sprint: SAND ↑ VJ: SAND ↑
	CONTROL	12	24.3 ± 2.1	81.2 ± 5.2	181.2 ± 4.4							

Hammami et al. 2020 [35]	RIGID	10	16.4 ± 0.5	69.7 ± 6.9	178.0 ± 0.7	7	3	21	45’	Lateral hurdle jumps	20-m sprint	20-m sprint: SAND ↑
	SAND	11	16.2 ± 0.6	70.8 ± 7.3	180.0 ± 0.3					Sprinting	Agility	Agility: SAND ↑
	CONTROL	10	16.5 ± 0.4	70.5 ± 5.7	179.0 ± 0.6					Horizontal jumps Hurdle jumps	VJ HJ	VJ: SAND ↑, RIGID ↑ HJ: ↔

Hammami et al. 2021 [41]	SAND	24	16.4 ± 0.4	71.0 ± 6.4	179.8 ± 3.1	7	-	-	-	Hurdle jumps HJ 10-m Sprint	V HJ 5-m sprint 10-m sprint 20-m sprint Agility Repeated sprint test	VJ: SAND ↑ HJ: = 5-m sprint: SAND ↑ 10-m sprint: SAND ↑ 20-m sprint: SAND ↑ Agility: SAND ↑ Repeated sprint test: SAND ↑
	CONTROL	18	16.2 ± 0.4	67.8 ± 4.9	170.4 ± 6.5							

Impellizzeri et al. 2007 [16]	GRASS	18	25 ± 4	74 ± 7	178 ± 7	4	3	12	NR	Vertical jumping	10-m sprint	10-m sprint: ↔
	SAND	17								Bounding Broad jumping DJ	20-m sprint VJ DOMS	20-m sprint: ↔ VJ: GRASS ↑, SAND ↑ DOMS: SAND ↑

Ojeda Aravena et al. 2022 [42]	RIGID	7	23.2 ± 2.1	72.1 ± 10.2	171.1 ±	4	3	12	NR	SJ with arms CMJA Slide skate Rebound jump Hurdles rebound jump Bulgarian squat jump	VJ	VJ: SOFT ↑
	SOFT	7	24.2 ± 3.2	74.5 ± 9.2	171.9 ± 5.2							

Ozen et al. 2017 [46]	GRASS	20	23.8 ± 3.4	73.0 ± 8.8	177.6 ± 4.33	3 yrs	2–3	NR	NR	NR	HJ	HJ: TARTAN ↑
	WOOD	12	21.58 ± 1.9	92.4 ± 10.8	192.4 ± 5.8						VJ	VJ: GRASS ↔
	TARTAN	12	23.92 ± 4.2	83.0 ± 7.74	189.8 ± 5.3						Muscular strength	Muscular strength: TARTAN ↑, WOOD ↑

Ozen et al. 2020 [36]	SAND WOOD	12	17.58 ± 0.504	87.7 ± 9.8	193.7 ± 7.0	6	3	18	NR	Vertical Jump Standing Long Jump Unilateral countermovement jumping 180 ° jumping repeated countermovement jumping board jumping drop jumps	HJ VJ Agility 30-m sprint	HJ: SAND ↑, WOOD ↑ VJ: SAND ↑, WOOD ↑ Agility: SAND ↑ 30-m sprint: SAND ↑

Pereira et al. 2023 [37]	SAND	7	18.5 ± 0.6	71.7 ± 5.0	178.6 ± 7.5	8	1	12	20–30	Bilateral hurdle jumps	VJ	VJ: ↔
	GRASS	8								Unilateral horizontal jumps Vertical drop jumps Horizontal drop jumps Linear sprint 90 º COD sprint	10-m sprint 17-m sprint CS Agility	10-m sprint: SAND ↑ 17-m sprint: SAND ↑ CS: SAND ↑, GRASS ↑ Agility: SAND ↑, GRASS ↑

Pereira et al. 2023 [17]	SAND	12	18.3 ± 0.5	69.2 ± 6.7	177.1 ± 7.2	6	2	12	50’	Skipping	VJ	VJ: SAND ↑, GRASS ↑
	GRASS	12								Alternate leg bounds Vertical bounding Bilateral hurdle jumps (frontal) Bilateral hurdle jumps (lateral) Linear sprint 90˚ COD sprint	Sprint Agility	Sprint: ↔ Agility: SAND ↑, GRASS ↑

Sporri et al. 2018 [38]	WATER	11	22.2 ± 2.5	77.7 ± 8.0	180 ± 0.8	8	3	24	NR	Jumping	VJ	VJ: WATER ↑
	CONTROL	9								Hopping Bounding	HJ 10-m sprint Agility	HJ: WATER ↑ 10-m sprint: ↔ Agility: WATER ↑

Vuong et al. 2023 [39]	SAND	9	26.7 ± 2.8	98.7 ± 14.0	194.3 ± 7.9	7	2	14	30–40	CoD exercises in combination with different plyometrics	VJ	VJ: SAND ↑↑, RIGID ↑
	RIGID	9	24.2 ± 4.6	88.5 ± 9.7	189.3 ± 8.7						HJ	HJ: SAND ↑↑↑, RIGID ↑
	CONTROL	7	22.6 ± 4.9	90.3 ± 9.3	194.4 ± 7.6						Sprint Agility	Sprint: SAND ↑↑↑, RIGID ↑ Agility: SAND ↑↑↑, RIGID ↑↑↑

BM = body mass, COD: change of direction, CS: curvilinear sprint, D = Duration, DJ: drop jump, DOMS: delayed onset muscle soreness, F = frencuency per week, HJ: horizontal jump, N = numbers, SJ = squat jump, T = total sessions, VJ: vertical jump, Wks = weeks

### Linear and curvilinear sprint speed

Twelve studies [16, 17, 32–41] included in this systematic review analyzed training surface and its corresponding effects on linear [16, 17, 32–41] and curvilinear sprint performance [37] (Table 1). Athletes in these studies were evaluated over sprint distances ranging from 5 to 60 meters, with only 1 study [37] also examining a curvilinear sprint of 17 meters. The intervention periods varied, lasting 8 weeks [32, 34, 37, 38], 7 weeks [33, 35, 39, 41], 6 weeks [15, 36, 40], and 4 weeks [14].

Seven studies [33, 35–37, 39–41] reported significant increases in linear sprint speed on sand. Five of these studies compared sand with firmer surfaces (i.e., grass [33, 37], gymnasium floor [35], wood [38], and parquet [39]), while 2 studies [40, 41] compared sand with a control group. Similarly, Pereira et al. [37] demonstrated that soccer players performing PT on sand for 8 weeks showed large improvements in curvilinear sprint speed (effects sizes of 1.28 and 1.46 for the right for the left sides, respectively). Notably, improvements were also observed in the group training on grass, with effect sizes of 1.10 for the right side and 0.98 for the left side. Moreover, 2 studies [32, 34] found significant positive effects on an aquatic surface compared to a firmer surface (i.e., mat [32], ground [34]) and a control group. Arazi et al., [32] observed significant improvements in both the water-based sprint group and the firm-ground sprint group (i.e., dry-land). Lastly, 3 studies [16, 17, 38] did not show any significant differences in this performance variable.

### Change of direction speed

Nine of the included studies [17, 33–39, 41] examined the effects of different surfaces on COD speed following distinct PT programs (Table 1). The tests included the Illinois and modified Illinois tests, modified t-test, repeated COD test, box agility test, modified zig-zag COD test, modified 5-0-5 test, and 5-10-5 test. The intervention periods ranged from 6 to 8 weeks (6 weeks [36, 37], 7 weeks [33, 35, 39, 41], and 8 weeks [17, 34, 38]).

Six studies [17, 35–37, 39, 41] demonstrated improvements for the group that trained on sand. These improvements were identified by comparing the outcomes with those of groups that trained on grass [17, 37], a “rigid” gym surface [35], wood [36], parquet [39], and a control group [41]. Chomani et al. [34] and Sporri et al. [38] found significant differences in favor of the group that trained in water. The former study [34] compared the water group with both a land-training group and a control group, while the latter [38] compared it solely with a control group. Lastly, only 1 study [33] found no significant differences between grass and sand surfaces.

### Vertical and horizontal jump performance

Sixteen studies [16, 17, 33–46] investigated the effects of PT on vertical and horizontal jump performance based on the type of surface (Table 1). The tests used to assess vertical and horizontal jump capabilities included SJ, CMJ, Abalakov jump (ABKJ), DJ, Sargent jump and five-jump tests, standing long jump test (one-footed and two-footed), and one-foot followed by a step test. The intervention periods ranged from 4 to 12 weeks (4 weeks [16, 42], 6 weeks [36, 37, 40], 7 weeks [33, 35, 39, 41], 8 weeks [17, 34, 38, 43], 10 weeks [44] and 12 weeks [45]), with 1 study using a 3-year protocol [46].

The results revealed that training on sand had significant positive effects on vertical jumping ability [16, 17, 33, 35, 36, 39–41, 45]. Furthermore, significant improvements in vertical jump performance were observed after PT programs conducted on rigid surfaces (e.g., parquet) [35, 39, 45], grass [16, 17], water [34, 38, 44], wood [36, 43], synthetic [43] and soft surfaces (e.g., carpet) [42]. Two studies [37, 46] reported no significant differences between groups. Specifically, one of them [37] compared training on sand and grass, while the other one [46] involved groups training on wood, tartan, and grass surfaces.

Regarding horizontal jumps, 3 studies [33, 36, 39] reported significant differences in favor of the group that trained on sand. Additionally, significant improvements in horizontal jump performance were observed following PT on water [34, 38], wood [36, 43], parquet [39], synthetic surfaces [43], and tartan [46]. However, 2 studies [35, 41] found no significant differences between the groups that trained on sand surface, on a rigid surface, and the control group (i.e., continued regular in season training).

### Muscular strength

Lower-limb muscular strength adaptations following PT completed on different surfaces were analyzed in 3 studies [29, 44, 46] (Table 1). While 2 studies employed medium-duration intervention protocols (i.e., 8-week and 10-week) [32, 44], the third study extended over a period of 3 years [46]. Cai et al. [44] found that performing PT in water for 10 weeks increased maximal dynamic strength compared to a control group that that did not undergo any training. Similarly, Arazi et al. [32] observed that, after an 8-week intervention, the group that trained on an aquatic surface showed greater improvements than the dry-land and control groups. Interestingly, Ozen et al. [46] found that training on firmer surfaces, such as wood and tartan, led to significantly better performance outcomes compared to training on a grass surface, which yielded the poorest results.

### Muscle damage and delayed onset muscle damage

Two studies [16, 44] included in this systematic review presented findings about the influence of PT surfaces on DOMS [16] or muscle damage [44] (Table 1). One study [16] used a 4-week intervention protocol with a frequency of 3 sessions per week, while another study [44] used a 10-week protocol with a frequency of 2 sessions per week. After 12 total sessions, the group that trained on sand reported less DOMS compared to the group that trained on grass [16], while the study comparing a group training in water to a control group found no significant differences on muscle damage (i.e., serum creatine kinase) or DOMS [44].

### Acute responses to plyometric training performed on different surfaces

Table 2 presents the results of studies that investigated the acute effects of PT programs conducted on different surfaces on neuromuscular performance. Of the 34 studies included in the systematic review, 11 examined the acute effects of various surfaces on performance outcomes. Among these, 2 studies measured sprint performance, 1 evaluated COD speed, and 11 assessed vertical and horizontal jumping ability, providing a comprehensive analysis of the immediate impact of these distinct surfaces on physical performance.

**TABLE 2:** Studies investigating the influence of training surface on acute responses induced by plyometric training.

Study	Study group	N	Age (years)	BM (kg)	Height (cm)	Test	Response
Arianasab et al. 2016 [50]	GRASS SYNTHETIC SAND RUBBER	20	21.8 ± 2.0	68.2 ± 5.0	178 ± 4.0	CMJ	VJ: SYNTHETIC ↑, RUBBER ↑
Bello et al. 2020 [49]	RIGID SAND	7 6	25.5 ± 2.8 22.0 ± 3	84.9 ± 5.1 83.5 ± 4.7	191.1 ± 1.3 188.0 ± 1.4	CMJ	VJ: ↔
Giatsis et al. 2004 [51]	RIGID SAND	15	25.6 ± 6.2	83.2 ± 6.0	188 ± 3.5	VJ	VJ: RIGID ↑
Giatsis et al. 2017 [52]	RIGID SAND	15	26.2 ± 5.9	83.4 ± 6.0	187 ± 0.5	VJ	VJ: RIGID ↑
Giatsis et al. 2022 [53]	RIGID SAND	16	26.2 ± 5.7	83.4 ± 5.8	187 ± 0.5	VJ	VJ: RIGID ↑
Malisoux et al. 2017 [54]	RIGID SOFT SOFTER MORE SOFTER SOFTEST	21	26.8 ± 5.7	79.9 ± 10.4	185 ± 7	VJ	VJ: RIGID ↑; SOFT ↑
Muramatsu et al. 2006 [55]	RIGID SAND	8	21.4 ± 0.7	72.6 ± 5.2	179.3 ± 6.0	VJ	VJ: ↔
Sanchez-Sanchez et al 2014 [48]	SURFACE 1 SURFACE 2 SURFACE 3 SURFACE 4	18	22.44 ± 1.72	73.74 ± 8.47	175 ± 6	RSA VJ	RSA: SURFACE 1 ↑, SURFACE 2 ↑ VJ: ↔
Sannicandro et al. 2023 [57]	GRASS SAND	20	16.6 ± 0.3	67.9 ± 4.7	179 ± 1.2	HJ	HJ: GRASS ↑
Streepey et al. 2000 [56]	WOOD COMPOSITE	21	20.5 ± 3.0	77.6 ± 7.9	182 ± 0.5	VJ	VJ: COMPOSITE ↑
Wannop et al. 2020 [47]	STIFF CONTROL SOFT SOFTER SOFTEST	17	26.4 ± 3.0	81.6 ± 11.1	181.9 ± 8.69	Agility VJ 10-m sprint	Agility: Softest ↑ VJ: Soft ↑ 10-m sprint: ↔

BM = body mass, D = Duration, DJ: drop jump, DOMS: delayed onset muscle soreness, F = frencuency per week, HJ: horizontal jump, N = numbers, RSA: repeated sprint ability, T = total sessions, VJ: vertical jump, Wks = weeks

### Linear sprint speed

Two studies [47, 48] investigated the acute effects of PT on linear sprint performance based on the type of surface (Table 2). In 1 study [47], participants performed a 10-meter sprint test on five different surfaces with varying levels of stiffness (i.e., stiff [+17%], control [0%], soft [-16%], softer [-34%], softest [-50%]), and no differences between surfaces were reported. In the other study [48], players completed a repeated sprint ability (RSA) test on 4 different surfaces (surface 1: compacted gravel sub-base without an elastic layer; surface 2: compacted gravel sub-base with an elastic layer; surface 3: asphalt sub-base without an elastic layer; and surface 4: asphalt sub-base with an elastic layer) with significant differences found in favor of surface 1 and 2.

### Change of direction speed

Only 1 study [47] analyzed the acute effects of PT performed on surfaces with different characteristics on COD ability (Table 2). The authors found significant improvements in COD performance (i.e., decreases in COD test time) in favor of the softest surface (i.e., with a 50% lower stiffness than the control condition) compared to the other 4 surfaces (i.e., softer, soft, control, stiff).

### Vertical and horizontal jump performance

Eleven studies [47–57] investigated the acute responses to PT on vertical [47–56] and horizontal [57] jumps depending on the type of surface (Table 2). CMJ, ABKJ, DJ and horizontal jumps were among the tests used to evaluate vertical and horizontal jump capabilities. The results revealed that rigid surfaces had significant positive effects on vertical jump performance [51–54]. Similarly, soft surfaces also elicited significant positive effects on vertical jump [47, 54]. Notably, 1 study [50] demonstrated significant differences in favor of artificial grass and rubber surfaces, while 3 others [48, 49, 55] observed no significant differences. Two of these studies [49, 55] compared a rigid surface with a sand one, while the remaining study [48] compared 4 different surfaces (surface 1: compacted gravel sub-base without an elastic layer, surface 2: compacted gravel sub-base with an elastic layer, surface 3: asphalt sub-base without an elastic layer, surface 4: asphalt sub-base with an elastic layer). Regarding the horizontal jump, 1 study [57] revealed significant differences in favor of the grass surface compared to sand surface.

### Footwear

Table 3 presents the results of the effects of different footwear on neuromuscular performance. Only investigations that analyzed acute responses were included. Of the 35 studies in the systematic review, 8 investigated the effects of different footwear on performance outcomes. All 8 studies measured vertical jump height, and 1 evaluated COD speed. Remarkably, no study involving barefooted PT was included.

**TABLE 3:** Studies investigating the influence of footwear type on acute responses induced by plyometric training.

Study	Study group	N	Age (yrs)	BM (kg)	Height (cm)	Test	Response
Bruce et al. 2019 [58]	VSS STS STND	29	19.1 ± 3.3	78.3 ± 12.2	190 ± 1.0	VJ	VJ: ↔
Chowning et al. 2020 [22]	MAX STND	21	23 ± 2	86.5 ± 15.4	179.8 ± 6.3	VJ	VJ: ↔
Firminger et al. 2019 [21]	VSS STS STND	30	18.9 ± 3.4	77.6 ± 12.7	190 ± 1.0	VJ	VJ: ↔
Jia et al. 2022 [23]	STS STND	15	21.2 ± 1.3	73.4 ± 5.6	176.7 ± 3.5	VJ	VJ: STS ↑
Lapole et al. 2013 [59]	STS STND	10	23.3 ± 5	75.2 ± 9.6	180 ± 7.5	VJ	VJ: STS ↑
Malisoux et al. 2017 [54]	MIN STND	21	26.8 ± 5.7	79.9 ± 10.4	185 ± 7	VJ	VJ: STND ↑
Teng et al. 2022 [60]	THICKEST THICK STND THIN THINNEST	15	23.7 ± 3.1	73.5 ± 4.7	178 ± 0.3	VJ 10-m sprint	VJ: THINNEST ↑, THIN ↑ 10-m sprint: THIN ↑
Worobets et al. 2015 [61]	STS STND THIN	20	NR	NR	NR	VJ 10-m sprint Agility	VJ: ↔ 10-m sprint: STS ↑ Agility: STS ↑

BM = body mass, CUSH = cushioned shoes, D = Duration, F = frencuency per week, MAX = maximally cushioned shoes, MIN = minimalist shoes, N = numbers, RFP = raised forefoot platforms, STND = standard cushioned shoes, STS = stiff shoes, T = total sessions, VJ = vertical jump, VSS = very stiff shoes, Wks = weeks

### Vertical jump performance

Eight studies [21–23, 54, 58–61] included in this review investigated the effects of PT using different footwear on vertical jump performance (Table 3). One study [54], comparing minimalist footwear and standard footwear (e.g., running shoes), found that the latter yielded better results than the former. In contrast, rigid footwear (i.e., stiff shoes) showed improvements in vertical jumping ability in 2 studies [23, 59], both compared to standard footwear. Another study [60] found significant improvements in COD performance (i.e., decreases in COD test time) in favor of the thinnest and thin footwear (i.e., with a 6 mm and 3 mm thinner midsole than the medium condition, respectively) compared to the other three types of footwear (i.e., medium, thick, and thickest). The remaining studies [21–23, 58, 61] did not show significant differences. Two of them [21, 58] compared 3 types of footwear (i.e., very stiff, stiff and standard shoes), 1 [22] compared 2 types of footwear (i.e., maximally cushioned shoes and standard cushioned shoes), and the remaining study [61] compared 3 types of footwear (i.e., stiff, standard and thin shoes).

### Change of direction speed

One study [61] included in this systematic review, which focused on footwear, made inferences about the effects of PT on COD performance (Table 3). After comparing 3 types of shoes (i.e., stiff [+20%], standard [0%], and thin shoes [-20%]), there was significant difference between stiff and thin shoes, with the stiffer shoes demonstrating a time reduction of 1.7%.

## DISCUSSION

The aim of this study was to synthesize and analyze the available evidence on the acute responses and neuromuscular adaptations generated by PT performed on different surfaces and using different types of footwear. Specifically, it aimed to examine how these factors can affect certain physical capabilities and the perception of muscle soreness, providing a more comprehensive understanding of the relationship between training surface, footwear, and athletic performance. Overall, the results of this systematic review indicate that the appropriate selection of training surface and footwear is a crucial aspect to be considered in PT programs, as it can significantly affect numerous variables (i.e., linear and curvilinear sprinting ability, vertical and horizontal jump performance, COD speed, muscular strength, and muscle soreness) that are directly related to sport-specific skills.

The nature of the surface on which PT is conducted significantly impacts jumping biomechanics, execution technique, and applied force [14]. In this context, a number of studies have found significant positive moderate to large effects of performing PT on sand [16, 17, 33, 35–37, 40, 41, 45]. One possible explanation for this phenomenon lies in the unique characteristics of sand surfaces compared to stiffer ones, such as grass or wood. According to the concept of stiffness, which refers to the resistance of an object to deformation in response to an applied force [62], energy tends to dissipate on sand surfaces due to their lower reaction to each contact made by the athlete. For instance, Bishop [63] reported that dry and uncompressed sand absorbs nearly 100% of the energy generated from ground impact. This type of surface is characterized by air gaps, causing displacement of the surface underfoot pressure with each movement [64]. As a result, muscle-tendon efficiency is lower compared to firmer surfaces, due to the increased energy consumption relative to the mechanical work performed [65]. This notion is supported by the study by Zamparo et al. [66], who demonstrated that running on sand increases energy expenditure by 15% to 40% compared to a firm surface. This additional energy expenditure may be attributed to the lower limb muscles performing extra work to stabilize the point of reaction on the surface [66]. Consequently, ground contact times are prolonged, resulting in a longer SSC [39], which favors greater development of the muscle’s contractile area. In contrast, on stiffer surfaces such as grass or wood, there is no substantial energy loss, and development primarily occurs at the muscletendon level. However, the increased demands of exercising on sand appear to promote superior or, at least, similar physical performance adaptations compared to firm surfaces.

Building on this, Rodriguez-Campillo et al. [18] suggested that for untrained individuals, PT on soft surfaces requires a high volume of contacts (i.e., 120 jumps per session or 240 jumps per week) to produce significant adaptations. In contrast, on firmer surfaces, where high-impact reaction forces are present, the required volume is lower (i.e., 60 jumps per session or 120 jumps per week). Thus, although training on sand appears to yield better adaptations than training on rigid surfaces, it is worth considering that the latter may require only half the number of contacts. Therefore, the increased efficiency of stiffer surfaces may promote adaptations in power-related capabilities in a more time-efficient manner (i.e., requiring less total training time). This is particularly relevant given the current challenge of limited time allocated for physical preparation in high-level sport settings, where congested fixture schedules increase the need for rapid adaptations [67]. Additionally, accessing surfaces like sand for PT is more challenging in the daily routine of team-sports compared to rigid surfaces (e.g., parquet, concrete, gym mats), which may present a disadvantage for conducting PT on sand. It is important to note that grass, despite often being considered a “rigid” surface, provides greater natural cushioning than parquet or gymnasium floors. Firmer surfaces (e.g., parquet), common in indoor sports facilities, are characterized by allowing more efficient energy transfer and maximizing energy return. This could potentially lead to more explosive movements and superior acute performance, due to the uniformity of the surface and minimal energy dissipation in parquet floors. Nonetheless, further research is needed to confirm these assumptions. From a practical perspective, the present results suggest that practitioners (particularly those working in soccer) may prescribe PT on the typical soccer field (i.e., grass) or in the gym (i.e., synthetic or wooden floor) to improve neuromuscular performance, with slight adjustments to training frequency and volume (i.e., number of sessions per week, jumps, or contacts) depending on the surface type.

Interestingly, 4 studies [32, 34, 38, 44] have reported superior improvements in certain variables (e.g., sprint speed, vertical and horizontal jump performance) when PT was performed in water, compared with dry-land PT [32, 34] and a control group [38, 44]. The buoyancy of water reduces the stretch reflex and the amount of eccentric loading during jumps [68]. However, due to the viscosity of water, the resistance during the concentric phase is greater than that experienced on land, resulting in aquatic PT potentially providing a distinct and effective stimulus [68]. This suggests that incorporating aquatic PT can be a valuable strategy for enhancing strengthpower performance, while minimizing joint and bone impact, as well as reducing eccentric muscle work. This makes it especially beneficial for athletes recovering from injuries or for those looking to diversify their training strategies while reducing the risk of injury.

Regarding the acute effects of PT on different surfaces, the results presented mixed outcomes. In terms of linear sprint performance, 1 study [47] found no significant differences between surfaces with varying levels of stiffness, while another study [48] suggested that certain rigid surfaces led to better sprint times. Similarly, improvements in COD ability were significantly associated with softer surfaces in 1 study [61], indicating their potentially superior benefits, although this was not consistently observed across all studies. Performance in vertical and horizontal jumps also showed varied results. Some studies [51–54] found significant positive moderate to large effects on vertical jump performance with rigid surfaces, likely due to their ability to facilitate energy transfer. Conversely, other studies [47, 50, 54] highlighted the benefits of softer surfaces or specific materials like artificial grass and rubber. However, in some cases [48, 49, 55], no significant differences were found between surfaces, indicating that surface type may not always be a critical factor. Overall, the diversity of results suggests that no definitive conclusion can be drawn about the superiority of different surfaces regarding the acute responses to PT.

The results of the current study indicate that footwear choice during training sessions is crucial, significantly affecting biomechanical dynamics, movement precision and technique, and force application. Three studies [23, 59, 61] included in this review, all of which investigated acute effects, reported improvements in vertical jump performance [23, 59] and COD and sprint [61] ability with stiffer footwear. According to Stefanyshyn and Nigg [69], each joint goes through phases of energy absorption and production. If this energy is dissipated instead of stored for later reuse, it can decrease athletic performance [69]. In the case of these studies [23, 59, 61], stiffer footwear appears to prevent energy loss, which may enhance sport-specific performance.

Malisoux et al. [54] examined differences in vertical jump performance between standard and minimalist shoes. They observed that standard shoes resulted in superior heights during ankle jumps and CMJ compared to minimalist shoes. Conversely, Teng et al. [60] reported better vertical jump and sprint performance with thinner-soled shoes (i.e., thinnest and thin) after comparing 5 types of footwear with different sole thicknesses (i.e., thinnest, thin, standard, thick, and thickest). Since the shoes differed only in sole thickness, with similar stiffness across all types, the improved performance with thinner soles suggests that the reduced material between the foot and the ground may have contributed to better results. Currently there is no robust evidence supporting the use of stiff, standard, or minimalist shoes. However, it is widely recognized that greater sole rigidity can prevent energy loss, thereby enhancing athletic performance [23, 68]. Since obtaining especially stiff or minimalist footwear may be challenging in some contexts, barefoot PT could serve as a potential solution, requiring no additional financial investment. Therefore, further research is warranted, since: 1) no studies have compared the mid- to long-term adaptations to PT performed with different footwear; and 2) the current literature does not provide sufficient evidence to confidently support the effectiveness of barefoot PT in athletic populations. This observation is somewhat surprising given the increasing anecdotal (but untested) evidence of this training strategy being used among athletes. Therefore, further research is warranted, as the current literature does not provide sufficient evidence to confidently support the effectiveness of barefoot PT in athletic populations.

Several limitations of this systematic review must be considered. Initially, this study was designed to synthesize and analyze the influence of surface type and footwear on neuromuscular adaptations following PT in professional soccer players. However, we encountered a significant scarcity of relevant literature in this population, prompting us to broaden our search to include team-sports in general. Although there is literature on the effects of footwear and surface on training, studies specifically focusing on PT in team-sports are scarce. Hence, we combined data from both youth and adult players (> 14 years old) to increase the sample size, which may have contributed to the heterogeneity of the findings. Consequently, the conclusions of this study should be interpreted with caution and confirmed by future research. Another key limitation is the use of a wide range of performance tests across the different studies (e.g., more than 8 COD tests with varying angles and velocity demands), which limits the robustness and generalization of the findings. Further research is encouraged to explore the effects of PT under different surfaces and footwear conditions – particularly barefoot, due to the limited evidence – as well as to investigate the underlying mechanisms responsible for the observed improvements in athletic performance. These additional studies could provide a more comprehensive understanding of how to optimize the effects of PT programs for various sports and athletes.

## CONCLUSIONS

This study demonstrates that PT performed on both soft (e.g., sand, water) and rigid (e.g., grass, parquet) surfaces, as well as using minimalist or rigid footwear, can lead to significant improvements in various independent measures of athletic performance. Specifically, sand surfaces appear to offer enhanced benefits for COD ability, jump performance, linear and curvilinear sprint speed, and muscular strength, while also reducing DOMS. However, rigid surfaces like grass and parquet may provide practical advantages in terms of time-efficiency, requiring lower PT volumes and fewer contacts to achieve comparable neuromuscular adaptations. The selection of the training surface and footwear should be carefully aligned with coaching objectives, the characteristics and needs of the athletes, and environmental constraints. For example, PT on grass or parquet might yield similar neuromuscular adaptations to training on sand, but with greater accessibility and efficiency. Minimalist or rigid footwear is also recommended, as they seem to offer superior (acute) responses when compared with cushioned shoes. Our findings emphasize the importance of considering both surface type and footwear when designing and prescribing PT, ensuring that they are tailored to maximize the desired adaptations in athletic performance. In summary, soft surfaces enhance neuromuscular recruitment and may reduce muscle soreness, while rigid surfaces offer greater time-efficiency and require lower PT volumes, although they may increase impact forces. Balancing these factors is essential for optimizing PT programs.

## Supplementary Material

Effects of plyometric training performed on different surfaces and with different types of footwear on the neuromuscular performance of team-sport athletes: A systematic review
