# Supplementary material for: Effects of plyometric training performed on different surfaces and with different types of footwear on the neuromuscular performance of team-sport athletes: A systematic review
Source: Biol Sport. 2025 Apr 28;42(4):107–20. doi: 10.5114/biolsport.2025.150037 (PMC12492346; doi:10.5114/biolsport.2025.150037)
Supplement: Effects of plyometric training performed on different surfaces and with different types of footwear on the neuromuscular performance of team-sport athletes: A systematic review [file JBS-42-4-56016-s1.pdf]

SUPPLEMENTARY MATERIAL

PEDro checklist evaluation for the included studies in the surface and intervention group.

Study	Nº1	Nº2	Nº3	Nº4	Nº5	Nº6	Nº7	Nº8	Nº9	Nº10	Nº11	Score	Study quality
Arazi et al. 2011 [32]	0	1	0	1	0	0	0	1	1	1	1	**6**	High
Balasas et al. 2018 [45]	1	0	0	0	0	0	0	1	1	1	1	**4**	Moderate
Bonavolonta et al. 2021 [33]	0	1	0	0	0	0	0	1	1	1	0	**4**	Moderate
Cai et al. 2022 [44]	0	1	0	0	0	0	0	1	1	1	1	**5**	Moderate
Chomani et al. 2021 [34]	0	0	0	0	0	0	0	1	1	1	1	**4**	Moderate
Çimenli et al. 2016 [43]	0	0	0	1	0	0	0	1	1	1	1	**5**	Moderate
De Villareal et al. 2024 [40]	0	1	0	1	0	0	0	1	1	1	1	**6**	High
Hammami et al. 2020 [35]	0	0	0	0	0	0	0	1	1	1	1	**4**	Moderate
Hammami et al. 2021 [41]	0	1	0	1	0	0	0	1	1	1	1	**6**	High
Impellizzeri et al. 2007 [16]	0	1	0	1	0	0	0	1	1	1	1	**6**	High
Ojeda Aravena et al. 2022 [42]	1	1	1	1	0	0	0	1	1	1	1	**7**	High
Ozen et al. 2017 [45]	0	0	0	0	0	0	0	1	1	1	1	**4**	Moderate
Ozen et al. 2020 [36]	0	1	1	1	0	0	0	1	1	1	1	**7**	High
Pereira et al. 2023 [37]	0	1	0	1	0	0	0	1	1	1	1	**6**	High
Pereira et al. 2023 [17]	0	1	0	1	0	0	0	1	1	1	1	**6**	High
Sporri et al. 2018 [38]	0	1	0	0	0	0	0	1	1	1	1	**5**	Moderate
Vuong et al. 2023 [39]	0	0	0	0	0	0	0	1	1	1	1	**4**	Moderate

Study	Arianasab et al. 2016 [50]	Bello et al. 2020 [49]	Giatsis et al. 2004 [51]	Giatsis et al. 2017 [52]	Giatsis et al. 2022 [53]	Malisoux et al. 2017 [54]	Muramatsu et al. 2006 [55]	Sanchez-Sanchez et al. 2014 [48]	Sannicandro et al. 2023 [57]	Streepey et al. 2000 [56]	Wannop et al. 2020 [47]
Was the research question or objective in this paper clearly stated?	Yes	Yes	Yes	Yes	Yes	Yes	Yes	Yes	Yes	Yes	Yes
Was the study population clearly specified and defined?	Yes	Yes	Yes	Yes	Yes	Yes	Yes	Yes	Yes	Yes	Yes
Was the participation rate of eligible persons at least 50%?	Yes	Yes	Yes	Yes	Yes	Yes	Yes	Yes	Yes	Yes	Yes
Were all the subjects selected or recruited from the same or similar populations (including the same time period)? Were inclusion and exclusion criteria for being in the study prespecified and applied uniformly to all participants?	No	No	No	Yes	Yes	Yes	No	No	No	No	No
Was a sample size justification, power description, or variance and effect estimates provided?	No	Yes	No	No	Yes	Yes	No	No	No	No	No

Study	Arianasab et al. 2016 [50]	Bello et al. 2020 [49]	Giatsis et al. 2004 [51]	Giatsis et al. 2017 [52]	Giatsis et al. 2022 [53]	Malisoux et al. 2017 [54]	Muramatsu et al. 2006 [55]	Sanchez-Sanchez et al. 2014 [48]	Sannicandro et al. 2023 [57]	Streepey et al. 2000 [56]	Wannop et al. 2020 [47]
For the analyses in this paper, were the exposure(s) of interest measured prior to the outcome(s) being measured?	No	No	No	No	No	No	No	No	No	No	No
Was the timeframe sufficient so that one could reasonably expect to see an association between exposure and outcome if it existed?	No	No	No	No	No	No	No	No	No	No	No
For exposures that can vary in amount or level, did the study examine different levels of the exposure as related to the outcome (e.g., categories of exposure, or exposure measured as continuous variable)?	No	No	No	No	No	No	No	No	No	No	No
Were the exposure measures (independent variables) clearly defined, valid, reliable, and implemented consistently across all study participants?	Yes	Yes	Yes	Yes	Yes	Yes	Yes	Yes	Yes	Yes	Yes
Was the exposure(s) assessed more than once over time?	NA	NA	NA	NA	NA	NA	NA	NA	NA	NA	NA
Were the outcome measures (dependent variables) clearly defined, valid, reliable, and implemented consistently across all study participants?	Yes	Yes	Yes	Yes	Yes	Yes	Yes	Yes	Yes	Yes	Yes
Were the outcome assessors blinded to the exposure status of participants?	No	No	No	No	No	Yes	No	No	No	No	No
Was loss to follow-up after baseline 20% or less?	Yes	Yes	Yes	Yes	Yes	Yes	Yes	Yes	Yes	Yes	Yes
Were key potential confounding variables measured and adjusted statistically for their impact on the relationship between exposure(s) and outcome(s)?	Yes	Yes	Yes	Yes	Yes	Yes	Yes	Yes	Yes	Yes	Yes
Quality rating (Good, fair, or poor)	Poor	Fair	Poor	Fair	Fair	Fair	Poor	Poor	Poor	Poor	Poor

Quality Assessment of Included Cross sectional using the NHLBI's Quality Assessment Tool for Observational Cohort and Cross-Sectional Studies

Study	Bruce et al. 2019 [58]	Chowning et al. 2020 [22]	Firminger et al. 2019 [21]	Jia et al. 2022 [23]	Lapole et al. 2013 [59]	Malisoux et al. 2017 [54]	Teng et al. 2022 [60]	Worobets et al. 2015 [61]
Was the research question or objective in this paper clearly stated?	Yes	Yes	Yes	Yes	Yes	Yes	Yes	Yes
Was the study population clearly specified and defined?	Yes	Yes	Yes	Yes	Yes	Yes	Yes	Yes
Was the participation rate of eligible persons at least 50%?	Yes	Yes	Yes	Yes	Yes	Yes	Yes	Yes
Were all the subjects selected or recruited from the same or similar populations (including the same time period)? Were inclusion and exclusion criteria for being in the study prespecified and applied uniformly to all participants?	Yes	No	No	No	No	Yes	No	No
Was a sample size justification, power description, or variance and effect estimates provided?	Yes	Yes	Yes	Yes	No	Yes	Yes	No
For the analyses in this paper, were the exposure(s) of interest measured prior to the outcome(s) being measured?	No	No	No	No	No	No	No	No
Was the timeframe sufficient so that one could reasonably expect to see an association between exposure and outcome if it existed?	No	No	No	No	No	No	No	No
For exposures that can vary in amount or level, did the study examine different levels of the exposure as related to the outcome (e.g., categories of exposure, or exposure measured as continuous variable)?	No	No	No	No	No	No	No	No
Were the exposure measures (independent variables) clearly defined, valid, reliable, and implemented consistently across all study participants?	Yes	Yes	Yes	Yes	Yes	Yes	Yes	Yes
Was the exposure(s) assessed more than once over time?	NA	NA	NA	NA	NA	NA	NA	NA
Were the outcome measures (dependent variables) clearly defined, valid, reliable, and implemented consistently across all study participants?	Yes	Yes	Yes	Yes	Yes	Yes	Yes	Yes
Were the outcome assessors blinded to the exposure status of participants?	No	No	No	No	No	Yes	No	No
Was loss to follow-up after baseline 20% or less?	Yes	Yes	Yes	Yes	Yes	Yes	Yes	Yes
Were key potential confounding variables measured and adjusted statistically for their impact on the relationship between exposure(s) and outcome(s)?	Yes	Yes	Yes	Yes	Yes	Yes	Yes	Yes
Quality rating (Good, fair, or poor)	Fair	Fair	Fair	Fair	Poor	Fair	Fair	Poor

Quality Assessment of Included Cross sectional using the NHLBI's Quality Assessment Tool for Observational Cohort and Cross-Sectional Studies
